# The influence of Baduanjin on sleep and depression and anxiety of college students with Qi-deficiency constitution: a randomized controlled trial

**DOI:** 10.3389/fspor.2026.1799065

**Published:** 2026-04-08

**Authors:** Ma Mengjuan, Deng Shuhong, Chen Yinan, Pan Jieling, Gong Zhichao, Li Wu, Li Jiangshan

**Affiliations:** 1 Acupuncture, Massage and Rehabilitation College, Hunan University of Traditional Chinese Medicine, Changsha, Hunan, China; 2 The Second Affiliated Hospital of Hunan University of Chinese Medicine, Changsha, Hunan, China

**Keywords:** Baduanjin, depression and anxiety, Pittsburgh Sleep Quality Index (PSQI), Qi-deficiency, sleep quality

## Abstract

**Introduction:**

Qi-deficiency constitution is a common constitution among college students and has already affected their normal life and study. Baduanjin promotes the circulation of Qi and Blood, and replenishes vital energy, which is of great significance for improving the improving Qi deficiency constitution of college students. This study aims to explore the clinical effects of a 10-week practice of Baduanjin exercise on the sleep quality and emotional state of college students with Qi-deficiency constitution.

**Methods:**

37 college students with Qi-deficiency constitution and sleep quality problems were randomly divided into 18 cases in the control group and 19 cases in the training group. We compared the Qi-deficiency constitution conversion score, Pittsburgh Sleep Quality Index (PSQI), Self-Rating Anxiety Scale (SAS) and Self-Rating Depression Scale (SDS) of the two groups before and after the intervention, and analyzed the correlation between the difference of the Qi-deficiency constitution conversion score and the difference of the total score of the PSQI as well as the clinical effectiveness of the two groups before and after the intervention.

**Results:**

Before the intervention, the two groups were comparable in terms of Qi-deficiency constitution conversion score, PSQI, SAS and SDS (*P* > 0.05). After the intervention, there was no significant difference in Qi-deficiency constitution conversion score, PSQI, SAS and SDS in the control group (*P* > 0.05); the training group's Qi-deficiency constitution conversion score, PSQI, SAS and SDS were lower than that of the group before the intervention (*P* < 0.05) and lower than that of the control group during the same period (*P* < 0.05). There was a significant positive correlation between the difference between the Qi-deficiency constitution conversion score and the difference between the total PSQI score before and after the intervention in the training group (*P* = 0.013). After the intervention, the total effective rate of the training group was higher than the control group (*P* < 0.001).

**Conclusion:**

Baduanjin can effectively improve the sleep quality, Qi-deficiency constitution and negative emotions of depression and anxiety in college students with Qi-deficiency constitution, and it is positively correlated with the improvement of Qi-deficiency constitution in terms of the degree of sleep improvement.

**Clinical Trial Registration:** identifier ITMCTR2025000103.

## 1 Introduction

Qi-deficiency constitution refers to the weak state caused by the lack of Qi in the human body, and the basic function of Qi is low. The main clinical manifestations are low voice, pale complexion, shortness of breath and lazy speech, often sweating, especially movement, weak pulse and so on (1). In a cross-sectional survey, the prevalence rate of Qi-deficiency constitution (53.90%) ranked first among the abnormal constitutions of the general population in China (2).

The heavy academic burden, the confusion in career choices, and the complexity of interpersonal relationships all have an impact on the immature psychology of college students, leading to sleep problems and negative emotions (3). Moreover, the excessive use of smartphones by college students can also affect their sleep patterns (4). The currently common treatment approach mainly involves the use of sleeping pills. However, long-term use can lead to dependence and addiction (5). Moreover, the co-occurrence rate of sleep disorders and depression/anxiety is relatively high (6). In the research on the physical constitution of patients with insomnia and the relationship between sleep quality and psychological conditions, it was found that the insomnia patients with Qi-deficiency constitution were more prone to develop negative emotions such as depression and anxiety (7, 8).

Baduanjin belongs to the Chinese Daoyin health exercise, which integrates the three regulation of “regulating the body”, “regulating the breath” and “regulating the heart” to dredging meridians, harmonizing Qi and Blood and achieving the unity of body and mind, strengthening the body, preventing and treating diseases (9, 10). Regular practice of Baduanjin exercises might be an effective and safe way to enhance the physical fitness related to health in young people (11). Our previous research has confirmed that practicing Baduanjin can effectively improve the Yang deficiency biased constitution and sleep quality of college students (12, 13). Based on this, this study explores the influence of Baduanjin on the sleep quality and mood of college students with Qi-deficiency constitution.

## 2 Material and methods

### 2.1 Study design

This study is a randomized, controlled, evaluator-blinded, two-arm parallel-group clinical trial. This research has been approved by the Research Ethics Committee of the Second Affiliated Hospital of Hunan University of Chinese Medicine, with the registration number 2024-KY-049. The study design was registered at International Traditional Medicine Clinical Trials Registry identifier (ITMCTR2025000103). This study was conducted in full accordance with the Helsinki Declaration of 1964. All participants read and signed the informed consent form before participating in this study. This study was conducted from March to July 2024.

### 2.2 Participants

The recruitment targets are students of Hunan University of Chinese Medicine. After filling out the “Classification and Judgment Form of Traditional Chinese Medicine Constitution” (14) and the “Pittsburgh Sleep Quality Index (PSQI)” (15), 37 individuals with Qi-deficiency constitution accompanied by sleep disorders were screened out. The inclusion criteria are as follows: (1) in the “Classification and Identification Table of Constitution in Traditional Chinese Medicine”, the conversion score of Qi-deficiency constitution is ≥40 points; (2) according to the “Pittsburgh Sleep Quality Index (PSQI)”, the total score of PSQI is ≥8 points; (3) students aged 18–30 from Hunan University of Traditional Chinese Medicine; (4) cooperating with this research protocol and signing the informed consent form. The exclusion criteria are as follows: (1) complicated with severe physical or mental diseases; (2) taking sedative-hypnotic/anti-anxiety and depression drugs in the past month; (3) being unable to cooperate with the research.

### 2.3 Randomization and evaluator-blind

This study adopted an evaluator-blinded single-blind design. For random sequence generation and allocation concealment, 40 eligible participants were divided into the training group and the control group by the random number table method. The participants were numbered 1–40 in two-digit format according to the recruitment sequence. The starting position was the fourth column of the second row of the random number table in the appendix of the textbook Statistics, and two-digit random numbers were read sequentially from left to right. Only the random numbers within the range of 1–40 were recorded, and those exceeding the range or repeated were discarded. The entire randomization process was completed by an independent third party not involved in the study to ensure the strictness of allocation concealment.

The evaluators and data analysts were kept blind to the group assignment of all participants. The trainers knew the grouping of the training group due to the particularity of Baduanjin training, but they did not participate in any other work such as outcome evaluation and data analysis, realizing the complete separation of trainers and evaluators. To reduce the subjective bias of participants on self-reported outcome indicators, all participants were informed that the study was to explore the effect of different health intervention measures on physical and mental health of college students, without disclosing the specific research hypothesis of Baduanjin intervention. For data confidentiality and anonymization, all participants were assigned unique anonymous numbers during data entry, and no personal identifying information was linked to the research data.

### 2.4 Sample size

The sample size was determined using the PASS software (2023), based on the data from previous studies that showed that Baduanjin improved sleep quality significantly. Previous studies, through yoga and Baduanjin exercises, significantly improved the sleep quality of college students. The post-intervention values were 4.93 ± 2.25 for the yoga group and 7.00 ± 2.17 for the Baduanjin group. With an *α* level of 0.05 and a *β* level of 0.8, the sample size was calculated to be 38 participants. Considering a 5% dropout rate, the final total sample size was determined to be 40 participants, divided into two groups (*n* = 20).

### 2.5 Intervention

The control group maintained their normal daily study and life, and was advised to avoid regular physical exercise during the study period. In addition, the control group participated in a weekly online health education group, with each health education session lasting 30 min and conducted by TCM physicians with intermediate and senior professional titles. The education content included TCM health maintenance methods for Qi-deficiency constitution, the development of scientific sleep habits for college students, and psychological adjustment skills for relieving anxiety and depression. The intervention lasted for 10 weeks in total. A special person was assigned to record the attendance rate of the subjects, and those with an attendance rate lower than 80% were reminded by phone to ensure the standardized implementation of the health education intervention.

The training group received the practice training of Baduanjin in addition to the control group. The training was based on the version of “Health Qigong-Baduanjin” released by the Chinese National Sports Administration. The entire training process was guided and led by instructors with over 2 years of experience in qigong, to ensure the standardization of the movements. Each time after the training, the participants signed in on the daily log sheet to ensure compliance Baduanjin consists of eight movements:

1. Raising Both Hands to Regulate the Triple Energizer
2. Drawing the Bow Left and Right Like Shooting a Hawk
3. Raising One Arm to Regulate the Spleen and Stomach
4. Looking Backwards to Relieve the Five Fatigues and Seven Injuries
5. Shaking the Head and Wagging the Tail to Clear Heart Fire
6. Holding the Feet with Both Hands to Strengthen the Kidneys and Lumbar Region
7. Clenching Fists and Glaring Fiercely to Boost Vitality
8. Raising and Lowering the Heels Seven Times to Alleviate All Ailments

Before the practice, a 5-min warm-up activity was conducted, and at the end, the concluding movements were performed with smooth abdominal breathing. In the first and second weeks, the training included teaching the techniques and practicing the reinforcing movements. In the following 8 weeks, they practiced the techniques continuously for 4 rounds each time, with a 2-min break between each round. Practice for 60 min each time, 4 times a week, for a total of 10 weeks.

### 2.6 Outcomes

#### 2.6.1 Clinical efficacy

The clinical efficacy is evaluated based on the total score of PSQI (16). After the intervention, the total score of PSQI decreases, and a total score of ≤5 is considered as a complete recovery; a total score of 5 < PSQI total score ≤10 is considered as marked improvement; a total score of 10 < PSQI total score ≤15 is considered as effective; a total score of PSQI after the intervention is lower than 15, or the total score remains unchanged or increases after the intervention, is considered as ineffective. The sum of complete recovery, marked improvement, and effectiveness is the total efficacy. The total efficacy rate = total number of effective cases/total number of cases × 100%.

#### 2.6.2 Qi-deficiency constitution conversion score

The subjects fill out the “Chinese Medicine Constitution Classification and Judgment Form” based on their own conditions, and add up the scores of each item to obtain the original score. [((Original score − Number of items)/(Number of items × 4)) × 100] = Conversion score. The lower the conversion score, the more significant the improvement of Qi-deficiency constitution.

#### 2.6.3 PSQI

The PSQI scale is divided into 7 factor scores: sleep quality, sleep onset time, sleep duration, sleep efficiency, sleep disorders, hypnotic drugs, and daytime functional impairment. The higher the score, the worse the sleep quality.

#### 2.6.4 SAS and SDS

SAS (17) can directly reflect the patient's subjective feelings of anxiety, while SDS (18) can directly show the patient's subjective feelings of depression. The scores of 20 items in each scale are added together to obtain the raw score. The raw score is multiplied by 1.25 and the integer part is taken as the standard score. Both scales have a 4-level scoring standard. The higher the score, the more severe the condition.

#### 2.6.5 Correlation between changes in Qi-deficiency constitution conversion score and PSQI score within the group

The difference in Qi-deficiency constitution conversion score s and the total PSQI score difference before and after the intervention were calculated for both groups, and then a correlation analysis was conducted on these two differences.

### 2.7 Statistical analysis

All statistical analyses were performed using SPSS 25.0 software. The Shapiro–Wilk test was used to evaluate the normality of measurement data. Normally distributed measurement data were expressed as mean ± standard deviation (*x̅* ± *s*) and compared using the independent-samples *t*-test; non-normally distributed data were presented as median [interquartile range, M (P_25_, P_75_)] and analyzed using nonparametric tests. Count data were described as cases [*n* (%)] and compared using the chi-square test. Correlation analysis was performed using Pearson correlation analysis. Ranked data were compared using the Wilcoxon rank-sum test. A value of *P* < 0.05 was considered statistically significant.

## 3 Results

### 3.1 Demographic data

**Figure 1:**
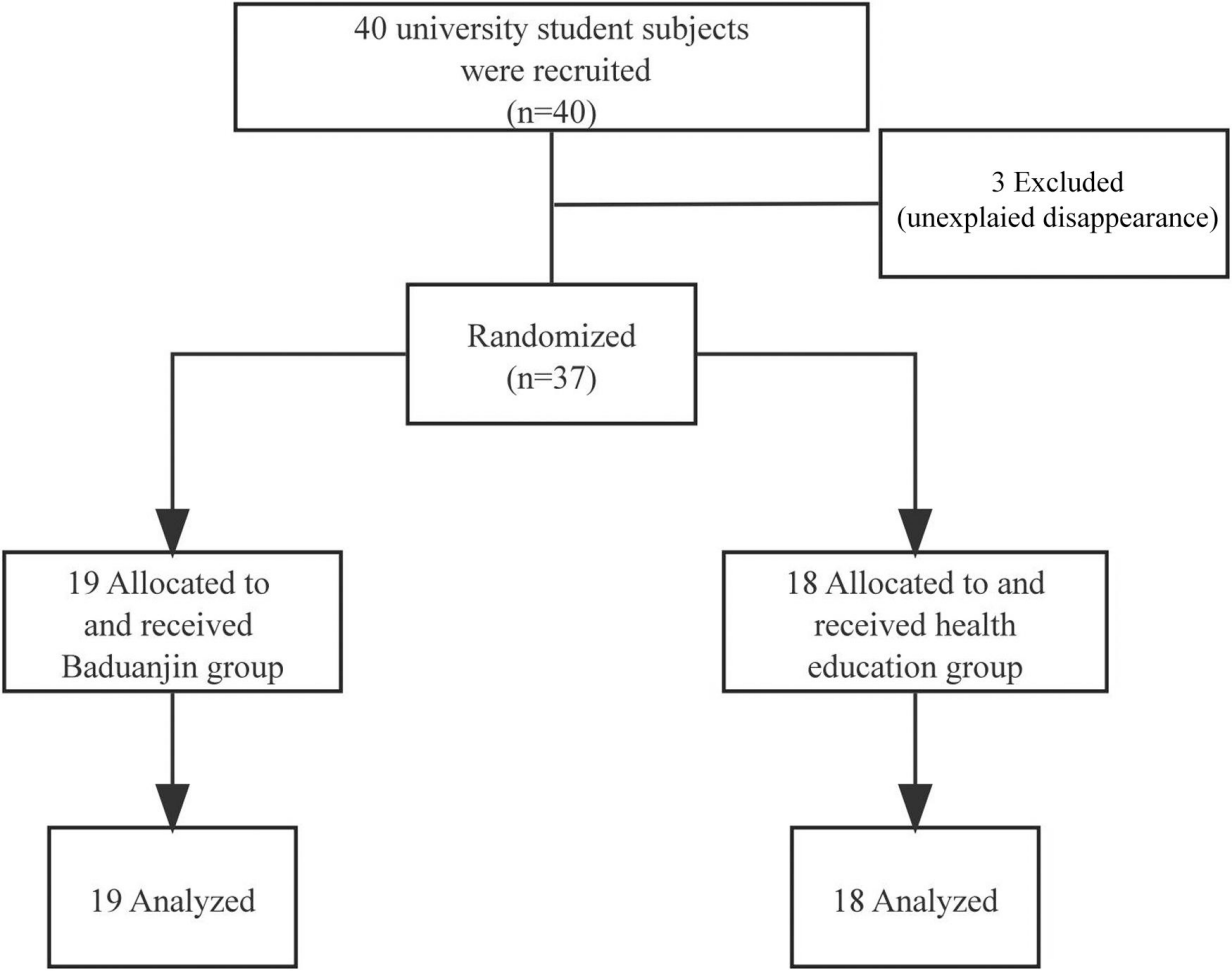
Consort.

Finally, 37 students from Hunan University of Chinese Medicine who met the inclusion criteria were recruited (3 cases were lost during the study). All of them agreed to participate in this study. They were divided into the training group (19 participants) and the control group (18 participants). Three students had no reason to quit. The CONSORT flowchart is included, As shown in Figure 1. There were no withdrawals due to adverse treatment reactions, and no complications occurred after the intervention. No significant differences were found in demographic characteristics between the training group and the control group see Table 1.

**Table 1:** Demographic characteristics of the study participants.

Project	TG	CG	*t/Z*	*P*
Sex(male/female)	1/18	1/17		0.374
Age(years)	19.95 ± 1.81	20.67 ± 2.40	−1.429	0.169
Weight(kg)	55.38 ± 8.09	54.31 ± 8.68	−0.334	0.753
Height(cm)	159.00 (156.00, 164.00	160.00 (156.50, 168.00)	−0.829	0.413

TG, training group; CG, control group.

Height was compared between groups using the Wilcoxon paired signed-rank test; age and weight were compared within groups using the Mann–Whitney *U* test. Normality of measurement data was tested by Shapiro–Wilk method, *W* and *P* values are presented in the Supplementary Material 1.

### 3.2 Comparison of clinical efficacy before and after the intervention of Baduanjin

After a 10-week randomized controlled trial, the total effective rate of the training group (94.74%) was higher than that of the control group (44.44%). The difference in clinical efficacy between the two groups was statistically significant (*P* < 0.001), as shown in Table 2.

**Table 2:** Clinical efficacy of the two groups after intervention [*n* (%)].

Group	*n*	Recovery	Marked improvement	Effective	Ineffective	Total efficacy
TG	19	9 (47.37)	9 (47.37)	0 (0.00)	1 (5.26)	94.74
CG	18	0 (0.00)	8 (44.44)	0 (0.00)	10 (55.56)	44.44
*x* ^2^						11.191
*P*						0.001

TG, training group; CG, control group.

Compared with before the intervention in this group. Clinical efficacy was ordinal categorical data, and inter-group comparison was performed using Mann–Whitney *U* test.

### 3.3 Qi-deficiency constitution conversion score

After the intervention, the conversion score of Qi-deficiency constitution in the training group (35.53 ± 19.07) was lower than that before the intervention in the same group (61.35 ± 10.53) and lower than that in the control group after the intervention (54.69 ± 18.39) (*P* < 0.05) see Table 3.

**Table 3:** Comparison of Qi-deficiency constitution conversion score the before and after intervention in the two groups (*x̅* ± *s*, scores).

Group	*n*	Before the intervention	After the intervention
TG	19	61.35 ± 10.53	35.53 ± 19.07**
CG	18	57.64 ± 13.53	54.69 ± 18.39
*t*		0.935	−3.108
*P*		0.356	0.004

TG, training group; CG, control group. Compared with before the intervention in this group. *P < 0.05, **P < 0.01, ***P < 0.01; compared with the control group, #*P* < 0.05, ##*P* < 0.01, ###*P* < 0.001. Both inter-group and intra-group comparisons were conducted using *t*-tests.

### 3.4 PSQI

After the intervention, the total PSQI score of the training group (10 points to 7 points) was lower than that of the control group after the intervention (10 points to 9 points). The sleep quality of the training group (2 points to 1 point) was lower than that of the control group after the intervention (2 points to 2 points), the sleep duration (1 point to 0 point) was lower than that of the control group after the intervention (1 point to 1 point), the sleep efficiency (1 point to 0 point) was lower than that of the control group after the intervention (1 point to 1 point), and the daytime functional impairment (2 points to 2 points) was also lower than that of the control group after the intervention (2 points to 2 points) see Table 4.

**Table 4:** Comparison of PSQI before and after intervention in the two groups [M (P_25_, P_75_), scores].

Project		CG	TG	*Z*	*P*
Sleep quality	Before	2.0 (1.0, 2.0)	2.0 (2.0, 2.0)	−0.645	0.599
	After	2.0 (1.75, 2.0)	1.0 (1.0, 1.0)**	−3.744	<0.001
Sleep onset time	Before	2.0 (2.0, 3.0)	2.0 (2.0, 3.0)	−0.050	0.964
	After	2.0 (1.75, 3.0)	1.0 (1.0, 2.0)	−1.230	0.245
Sleep duration	Before	1.0 (1.0, 2.0)	1.0 (0.0, 2.0)	−0.262	0.822
	After	1.0 (1.0, 1.0)	0.0 (0.0, 1.0)*	−2.583	0.019
Sleep efficiency	Before	1.0 (0.0, 2.0)	1.0 (0.0, 2.0)	−0.467	0.663
	After	1.0 (0.0, 1.0)	0.0 (0.0, 1.0)*	−2.398	0.031
Sleep disorders	Before	1.5 (1.0, 2.0)	1.0 (1.0, 2.0)	−0.137	0.916
	After	1.0 (1.0, 2.0)	1.0 (1.0, 1.0)	−1.764	0.150
Hypnotic drugs	Before	0.0 (0.0, 0.0)	0.0 (0.0, 0.0)	−0.973	0.799
	After	0.0 (0.0, 0.0)	0.0 (0.0, 0.0)	−0.039	0.988
Daytime dysfunction	Before	2.0 (1.75, 3.0)	2.0 (2.0, 3.0)	−0.496	0.663
	After	2.0 (2.0, 3.0)	2.0 (1.0, 2.0)*	−2.349	0.026
PSQI score	Before	10.0 (9.0, 11.0)	10.0 (9.0, 11.0)	−0.186	0.869
	After	9.0 (8.0, 11.0)	7.0 (4.0, 8.0)**	−3.820	<0.001

TG, training group; CG, control group. Data are presented as M (P_25_, P_75_). Within-group comparisons were performed using Wilcoxon signed-rank test; between-group comparisons using Mann–Whitney U test.

CG (*n* = 18); TG (*n* = 19). Compared with before the intervention in this group, *P < 0.05, **P < 0.01, ***P < 0.01; compared with the control group, #*P* < 0.05, ##*P* < 0.01, ###*P* < 0.001.

### 3.5 SAS, SDS

After the intervention, the anxiety score of the training group (42.24 ± 7.13) was lower than that before the intervention in this group (51.18 ± 8.18) and lower than that of the control group after the intervention (50.28 ± 8.84). The depression score of the training group after the intervention (45.46 ± 9.56) was lower than that before the intervention in this group (56.64 ± 6.36) and lower than that of the control group after the intervention (54.58 ± 12.88) (*P* < 0.05) see Table 5.

**Table 5:** Comparison of SAS and SDS scores before and after intervention in Two groups (*x̅* ± *s*, scores).

Group	*n*	SAS		SDS	
		Before	After	Before	After
TG	19	51.18 ± 8.18	42.24 ± 7.13**	56.64 ± 6.36	45.46 ± 9.56**
CG	18	49.03 ± 7.31	50.28 ± 8.84	53.82 ± 10.18	54.58 ± 12.88
*t*		0.844	−3.053	1.019	−2.456
*P*		0.404	0.004	0.315	0.019

TG, training group; CG, control group.

Compared with before the intervention in this group, *P < 0.05,

** *P* < 0.01, ***P < 0.01; compared with the control group, #*P* < 0.05, ##*P* < 0.01, ###*P* < 0.001. Both inter-group and intra-group comparisons were conducted using *t*-tests.

### 3.6 Correlation between changes in Qi-deficiency constitution conversion score and PSQI score within the group

The differences in the Qi-deficiency constitution conversion scores and the total PSQI scores before and after the intervention were calculated separately for the two groups. Then, a correlation analysis was conducted on the differences of the two groups. There was no correlation between the two differences in the control group (*P* > 0.05), while there was a significant positive correlation between the two differences in the training group (*P* < 0.05) see Table 6.

**Table 6:** Correlation between changes in Qi-deficiency constitution conversion score and PSQI score within the group [M (P_25_, P_75_), scores].

Group	*n*	Difference in transformation from Qi-deficiency constitution	Total score difference of PSQI	*r*	*P*
TG	19	25.00 (12.50, 40.62)	5.00 (2.00, 6.00)	0.768	0.013
CG	18	0.00 (−6.25, 7.03)	0.50 (−1.00, 1.00)	−0.332	0.178

TG, training group; CG, control group. Data are presented as M (P_25_, P_75_). The difference values did not conform to normal distribution by Shapiro–Wilk test, so Spearman rank correlation analysis was used.

## 4 Discussion

This study conducted a 10-week randomized controlled trial to explore the effects of Baduanjin practice on sleep quality, anxiety and depression in college students with Qi-deficiency constitution, and further analyzed the correlation between the improvement of Qi-deficiency constitution and sleep quality. The results consistently demonstrated that compared with simple health education, Baduanjin practice combined with health education significantly reduced the Qi-deficiency constitution conversion score, PSQI, SAS and SDS scores of college students with Qi-deficiency constitution, and the total effective rate of sleep improvement was significantly higher in the training group. Meanwhile, there was a significant positive correlation between the improvement degree of Qi-deficiency constitution and the amelioration of sleep quality in the TG, which confirmed the multi-dimensional beneficial effects of Baduanjin on this population and the internal link between its regulatory effects on constitution and sleep. As a widely spread traditional Chinese moderate-intensity aerobic exercise, Baduanjin is easy to learn and practice without time and space restrictions, making it a feasible intervention method for college students (19).

A core finding of this study is that Baduanjin can effectively ameliorate the Qi-deficiency constitution of college students, and its mechanism is closely related to the synergy of body, breath and mind regulation, which directly targets the TCM pathogenesis of Qi deficiency leading to dysfunction of the spleen in transportation and transformation, and malnourishment of the heart spirit. In terms of breath regulation, Baduanjin requires strict coordination of diaphragmatic breathing with body movements—abdominal contraction during exhalation and expansion during inhalation—which effectively enhances the respiratory capacity of the diaphragm and thoracoabdominal region (20). Improves pulmonary ventilation and cardiopulmonary function (21, 22) and lays a physical foundation for Qi generation in the human body. For body regulation, the gentle and targeted movements of Baduanjin, Raising One Arm to Regulate the Spleen and Stomach can stimulate the meridians and collaterals, promote the transportation and transformation function of the spleen and stomach—the root of Qi and blood generation—and thus alleviate the fundamental state of Qi deficiency caused by insufficient source of Qi production (23). The combination of regulated body and breath effectively promotes the generation and unobstructed circulation of Qi and blood in the body, gradually reversing the weak state of Qi-deficiency constitution.

The improvement of Qi-deficiency constitution by Baduanjin is the key link connecting the amelioration of sleep quality and negative emotions, forming a mutually reinforcing regulatory effect. On the one hand, the recovery of normal Qi and blood circulation and the improvement of spleen and stomach function after the amelioration of Qi-deficiency constitution can adequately nourish the heart spirit, which directly relieves the insomnia, poor sleep continuity and daytime functional impairment caused by heart spirit malnourishment—this is also the reason for the significant positive correlation between the improvement of Qi-deficiency constitution and sleep quality in the training group. On the other hand, the gentle and slow movements of Baduanjin reduce the body's excessive metabolic burden (24), and the stable breathing rhythm calms the disturbed mind, which together improve multiple dimensions of sleep including sleep quality, duration and efficiency in college students. This result is consistent with previous studies that confirmed Baduanjin's positive effect on sleep quality (25), and further expands its application scope from the elderly to young college students with Qi-deficiency constitution, suggesting that Baduanjin's sleep-improving effect has population universality, and its improvement of sleep quality is also conducive to protecting the cognitive health of college students in the growth and learning stage (26, 27).

In terms of relieving anxiety and depression, the regulatory effect of Baduanjin is also based on the improvement of Qi-deficiency constitution and the three-regulation synergy of body, breath and mind. TCM holds that negative emotions such as anxiety and depression in Qi-deficiency constitution population are often accompanied by stagnation of Qi movement on the basis of insufficient Qi, and the root cause is still the malnourishment of heart spirit due to insufficient Qi and blood (28). Before and during Baduanjin practice, practitioners are guided to relax the mind, keep calm and balance movement and stillness (20, 29), which directly soothes the disturbed emotions; at the same time, the improvement of Qi-deficiency constitution reverses the state of insufficient Qi and blood, nourishes the heart spirit, and fundamentally eliminates the physical basis of negative emotions. In addition, the regulation of Qi and blood circulation by Baduanjin relieves Qi stagnation, restores the normal functional state of the zang-fu organs (28), and forms a two-way regulation of body and mind. This is consistent with the conclusion of previous studies that Qigong-based exercises can improve emotional states (30), and further clarifies the targeted regulatory effect of Baduanjin on negative emotions in college students with Qi-deficiency constitution.

The integrated effect of Baduanjin on Qi-deficiency constitution, sleep quality and emotional state is essentially realized through the organic combination of body regulation, breath regulation and mind regulation—the core connotation of traditional Chinese Daoyin health exercise. Body regulation is the foundation: through standardized movements, it promotes the transportation and transformation of the spleen and stomach, unblocks the meridians, and lays a physical foundation for Qi generation and circulation; breath regulation is the key: through diaphragmatic breathing, it enhances cardiopulmonary function, coordinates the movement of Qi, and connects the body and mind; mind regulation is the guidance: through calming the mind and focusing attention, it relieves emotional tension, and makes the body and breath regulation more effective. The three aspects interact and complement each other, realizing the harmony of body and mind, restoring the normal flow of internal Qi, harmonizing the zang-fu organs, ensuring the source of Qi and blood generation, and thus simultaneously alleviating the symptoms of Qi-deficiency constitution, sleep disorders, anxiety and depression (31, 32). As a non-pharmaceutical intervention method with the effect of nourishing both body and spirit, Baduanjin has unique advantages in the health management of college students with Qi-deficiency constitution.

## 5 Conclusion

After practicing Baduanjin for 10 weeks, the scores of Qi-deficiency constitution conversion score, PSQI, SAS and SDS of the training group were significantly lower than those of the group before the intervention and the control group at the same period. Moreover, the improvement of sleep quality in the training group was also positively correlated with the improvement of Qi-deficiency constitution biased constitution *(P* < 0.05). This indicates that the practice of Baduanjin exercises combined with publicity and education can improve the Qi-deficiency constitution imbalance constitution, sleep disorders, anxiety and depression of college students, and the effect is superior to that of publicity and education alone. This provides a new idea for clinical treatment of patients with Qi-deficiency constitution and insomnia. As a moderate-intensity aerobic exercise, Baduanjin is easy to learn and flexible, and it is not restricted by time and space (19). It is suitable for people of all ages to practice. Through this exercise method, not only can the body's physical condition be enhanced, and the Qi-deficiency constitution and imbalance constitution be improved, but it also helps to improve sleep quality and alleviate depression and anxiety. It is of great significance for the physical and mental health development of college students.

This study is the first to target college students with Qi-deficiency constitution for Baduanjin intervention, filling the research gap compared with previous studies focusing on the elderly or college students with Yang-deficiency constitution; through correlation analysis, it reveals the clear action logic of “regulating constitution—improving sleep—relieving emotions”, providing direct evidence for the synergistic regulatory effect of Baduanjin on physical and mental health; and it designs a standardized, campus-adapted Baduanjin intervention scheme that is easy to popularize and operate without time and space restrictions. These innovations provide a safe non-pharmaceutical intervention for improving college students’ Qi-deficiency constitution, sleep quality and emotional state, avoiding the risk of drug dependence from long-term use of hypnotic or anti-anxiety drugs; promote the precise development of physical health management in universities by integrating TCM constitution screening with targeted Baduanjin intervention; enrich the forms of physical and mental health education in colleges and universities, offering a new effective exercise option for campus health promotion.

This study shows that while Baduanjin exercise can improve sleep quality, duration, alleviate sleep difficulties and daytime functional disorders, its improvement on sleep onset time is not significant, which may be due to: short 10-week intervention period failing to reverse long-term irregular sleep rhythms caused by academic tasks and smartphone overuse; daytime training unable to alleviate pre-sleep anxiety; uncontrollable collective dormitory environment interference; lack of objective PSG measurement. Additionally, the trial has notable limitations including an extremely small sample size, a single-center design that leads to potential selection bias and poor generalizability, a lack of long-term follow-up that fails to evaluate the sustainability of intervention effects, and an over-reliance on subjective sleep indicators that are prone to bias; to address these issues, future research should add 3/6/12-month follow-up to evaluate sustained effects and compliance factors, integrate objective indicators such as PSG, design different intensity/frequency/cycle groups to explore optimal intervention schemes, adopt stratified randomization and covariance analysis to exclude interference from diet, work and rest.

## Data Availability

The raw data supporting the conclusions of this article will be made available by the authors, without undue reservation.
