# Supplementary material for: The influence of Baduanjin on sleep and depression and anxiety of college students with Qi-deficiency constitution: a randomized controlled trial
Source: Front Sports Act Living. 2026 Apr 8;8:1799065. doi: 10.3389/fspor.2026.1799065 (PMC13099293; doi:10.3389/fspor.2026.1799065)
Supplement: Supplementary file 1 [file Datasheet1.pdf]

Supplementary Table 1.

Results of the Shapiro - Wilke normality test for general data

Variable	Group	W statistic	*P* value
Age(years)	TG	0.864	0.011
	CG	0.840	0.006
Weight(kg)	TG	0.939	0.257
	CG	0.842	0.006
Height(cm)	TG	0.934	0.207
	CG	0.917	0.116

TG, training group; CG, control group

Table 2

Results of the Shapiro-Wilk normality test for Qi-deficiency constitution the conversion scores before and after the intervention in the two groups

Variable	Group	W statistic	*P* value
Before the intervention	TG	0.948	0.371
	CG	0.934	0.225
After the intervention	TG	0.952	0.434
	CG	0.952	0.462

TG, training group; CG, control group

Table 3

Project		Group	W	P
Sleep quality	before	TG	0.778	0.001
		CG	＜0.001	＜0.001
	after	TG	0.626	＜0.001
		CG	0.373	＜0.001
Sleep onset time	before	TG	0.814	0.002

		CG	0.807	0.002
	after	TG	0.883	0.024
		CG	0.848	0.008
Sleep duration	before	TG	0.828	0.003
		CG	0.609	<0.001
	after	TG	0.747	<0.001
		CG	0.798	0.001
Sleep efficiency	before	TG	0.885	0.026
		CG	0.877	0.024
	after	TG	0.745	<0.001
		CG	0.786	0.001
Sleep disorders	before	TG	0.733	<0.001
		CG	0.843	0.007
	after	TG	0.778	0.001
		CG	0.662	<0.001
Hypnotic drugs	before	TG	0.244	<0.001
		CG		
	after	TG	0.244	<0.001
		CG	0.253	<0.001
Daytime dysfunction	before	TG	0.770	<0.001
		CG	0.786	0.001
	after	TG	0.873	0.016
		CG	0.801	0.002
PSQI score	before	TG	0.920	0.115
		CG	0.840	0.006
	after	TG	0.896	0.041
		CG	0.914	0.099

TG, training group; CG, control group

Table 4

Shapiro-Wilk test results of SAS and SDS before and after intervention in both groups

Project		Group	W	*P*
SAS	before	TG	0.925	0.140
		CG	0.954	0.493
	after	TG	0.943	0.305
		CG	0.917	0.116
SDS	before	TG	0.941	0.277
		CG	0.932	0.207
	after	TG	0.944	0.313
		CG	0.963	0.667

TG, training group; CG, control group
